# Mapping genetic variations to three-dimensional protein structures to enhance variant interpretation: a proposed framework

**DOI:** 10.1186/s13073-017-0509-y

**Published:** 2017-12-18

**Authors:** Gustavo Glusman, Peter W. Rose, Andreas Prlić, Jennifer Dougherty, José M. Duarte, Andrew S. Hoffman, Geoffrey J. Barton, Emøke Bendixen, Timothy Bergquist, Christian Bock, Elizabeth Brunk, Marija Buljan, Stephen K. Burley, Binghuang Cai, Hannah Carter, JianJiong Gao, Adam Godzik, Michael Heuer, Michael Hicks, Thomas Hrabe, Rachel Karchin, Julia Koehler Leman, Lydie Lane, David L. Masica, Sean D. Mooney, John Moult, Gilbert S. Omenn, Frances Pearl, Vikas Pejaver, Sheila M. Reynolds, Ariel Rokem, Torsten Schwede, Sicheng Song, Hagen Tilgner, Yana Valasatava, Yang Zhang, Eric W. Deutsch

**Affiliations:** 1 Institute for Systems Biology, Seattle, WA 98109, USA; 2 San Diego Supercomputer Center, University of California San Diego, La Jolla, CA 98093 USA; 3 RCSB Protein Data Bank, University of California San Diego, La Jolla, CA 98093 USA; 4 Human Centered Design & Engineering, University of Washington, Seattle, WA 98195 USA; 5 Division of Computational Biology, School of Life Sciences, University of Dundee, Dundee, DD1 5EH UK; 6 Department of Molecular Biology and Genetics, Aarhus University, 8000 Aarhus, Denmark; 7 Department of Biomedical Informatics and Medical Education, University of Washington, Seattle, WA 98109 USA; 8 University of California San Diego, La Jolla, CA 92093 USA; 9 Institute of Molecular Systems Biology, ETH Zurich, CH-8093 Zurich, Switzerland; 10 Institute for Quantitative Biomedicine, Rutgers, The State University of New Jersey, Piscataway, NJ 08854 USA; 11 Kravis Center for Molecular Oncology, Memorial Sloan Kettering Cancer Center, New York, NY 10065 USA; 12 SBP Medical Discovery Institute, La Jolla, CA 92037 USA; 13 AMPLab, University of California, Berkeley, CA 94720 USA; 14 Human Longevity, Inc, San Diego, CA 92121 USA; 15 Department of Biomedical Engineering, Institute for Computational Medicine, Johns Hopkins University, Baltimore, MD 21218 USA; 16 Department of Oncology, Johns Hopkins Medicine, Baltimore, MD 21287 USA; 17 Flatiron Institute, Center for Computational Biology, Simons Foundation, New York, NY 10010 USA; 18 Department of Biology and Center for Genomics and Systems Biology, New York University, New York, NY 10003 USA; 19 SIB Swiss Institute of Bioinformatics and University of Geneva, CH-1211 Geneva, Switzerland; 20 Institute for Bioscience and Biotechnology Research, University of Maryland, Rockville, MD 20850 USA; 21 Department of Cell Biology and Molecular Genetics, University of Maryland, College Park, MD 20742 USA; 22 Department of Computational Medicine & Bioinformatics, University of Michigan, Ann Arbor, MI 48109-2218 USA; 23 School of Life Sciences, University of Sussex, Brighton, BN1 9QG UK; 24 The University of Washington eScience Institute, Seattle, WA 98195 USA; 25 SIB Swiss Institute of Bioinformatics and Biozentrum University of Basel, CH-4056 Basel, Switzerland; 26 Brain and Mind Research Institute, Weill Cornell Medicine, New York City, NY 10021 USA

## Abstract

The translation of personal genomics to precision medicine depends on the accurate interpretation of the multitude of genetic variants observed for each individual. However, even when genetic variants are predicted to modify a protein, their functional implications may be unclear. Many diseases are caused by genetic variants affecting important protein features, such as enzyme active sites or interaction interfaces. The scientific community has catalogued millions of genetic variants in genomic databases and thousands of protein structures in the Protein Data Bank. Mapping mutations onto three-dimensional (3D) structures enables atomic-level analyses of protein positions that may be important for the stability or formation of interactions; these may explain the effect of mutations and in some cases even open a path for targeted drug development. To accelerate progress in the integration of these data types, we held a two-day Gene Variation to 3D (GVto3D) workshop to report on the latest advances and to discuss unmet needs. The overarching goal of the workshop was to address the question: what can be done together as a community to advance the integration of genetic variants and 3D protein structures that could not be done by a single investigator or laboratory? Here we describe the workshop outcomes, review the state of the field, and propose the development of a framework with which to promote progress in this arena. The framework will include a set of standard formats, common ontologies, a common application programming interface to enable interoperation of the resources, and a Tool Registry to make it easy to find and apply the tools to specific analysis problems. Interoperability will enable integration of diverse data sources and tools and collaborative development of variant effect prediction methods.

**Electronic supplementary material:**

The online version of this article (doi:10.1186/s13073-017-0509-y) contains supplementary material, which is available to authorized users.

## Background

Recent progress in DNA-sequencing technologies has ushered in an era of rapid and cost-effective genome sequencing, enabling clinical applications [1] and the potential for personalized systems medicine [2] through the understanding of an individual’s genetic risks and by integration with longitudinal phenotype measurements [3].The detailed knowledge of an individual’s genotype poses a significant interpretation challenge: while genetic variants disrupting transcript structure and protein-coding sequences (for example, nonsense mutations) have long been considered “low hanging fruit” relative to variants in non-coding sequences, the field still struggles with interpreting missense mutations, which are more common, and more frequently associated with disease [4]. This has led to an increasing number of variants of uncertain significance (VUS). To address the resulting annotation and reporting challenges [5, 6], the American College for Genetics and Genomics (ACMG) and the Association for Molecular Pathology (AMP) have released variant interpretation guidelines based on pathogenicity [7]. The interpretation of variants relies on a combination of multiple lines of evidence, including the frequency of the variant in the population (common variants are less likely to be pathogenic), the mode of segregation in pedigrees (for example, de novo mutations not observed in parents are more likely to be pathogenic than those that are inherited), the mode of presentation in affected individuals (for example, single dominant variant, single variant in homozygous state, two variants in compound heterozygous state), the predicted effect on RNA and protein sequence and structure, and prior knowledge accumulated in curated databases. Many computational tools have been developed to support these assessments (Additional file 1: Table S1). However, multiple challenges remain in the rapidly evolving field of clinical variant interpretation, including differences in allele frequency among different populations, a growing but still incomplete understanding of how variants affect gene regulation, the sequence and structure of RNA and protein products, and the partial, inconsistently presented and sometimes conflicting knowledge in databases.

To assess the potential pathogenicity of genetic variants, singly or in combinations, it is useful to assess their frequency in control or general populations, as already mentioned. Public databases are burgeoning with information about genetic variants in humans and in many model organisms. Resources such as dbSNP [8], dbVar [9], COSMIC [10], cBioPortal [11], UniProt [12], Kaviar [13], Clinvar [14], HGMD [15], ExAC, and gnomAD [16] provide data on hundreds of millions of single-nucleotide variants (SNVs) and other types of genetic variations. Each database has a different focus, different sources of data, processing methods, level of coverage, and degree of metadata associated with each variation; some focus only on human variation, while others cover many species. Similarly, each database has differing mechanisms for data access and differing levels of cross-referencing.

The biomedical research community is fortunate to have access to such a wealth of information, but its sheer size and disparate nature are also daunting. In addition to public databases, hundreds of DNA- and RNA-sequencing experiments are revealing manifold genetic variants and mutations each year, and an increasing number of these can be linked to protein structure. For example, protein structure analysis of a novel variant in the ubiquitin-protein ligase TRIM11, observed in individuals affected with inflammatory bowel disease, helped determine that the variant is more likely to affect protein–protein interactions rather than protein folding and stability [17]. Functionally important somatic variants in cancer may form statistically significant spatial clusters in three-dimensional protein structure, which are not detectable in one-dimensional sequence, such as kidney-cancer-specific variants in the tumor suppressor gene *VHL*, which are proximal to the binding site of VHL for its ubiquitination target HIF1A [18].

Simultaneously, there has been great progress in characterizing the 3D structures of proteins [19, 20], both experimentally and computationally. Essentially, all publicly available experimentally derived structures are deposited in the Protein Data Bank (PDB) [21]. When experimentally determined structures are not available for proteins, structural models may be used instead. Protein Model Portal [22] aggregates precomputed models from multiple resources, whereas most methods generate models interactively on request, for example, I-TASSER [23], ModWeb [24], Phyre2 [25], HHpred [26], or SWISS-MODEL [27]. Currently available homology models with 40–50% sequence identity to experimental structures already cover approximately 40% of the residues in the human proteome [28], although this does not always include the full-length protein in the correct quaternary structure, but often only specific domains. Beyond simply having 3D models of proteins, it is crucial to annotate the functional substructures in these models with such information as the locations of ligand-binding and active sites, functional domains, regions that are externally accessible versus in the protected interior, protein–protein interaction interfaces, and other structural features that might be related to function [29].

However, the connections between genetic variations and protein structure are not always easy to find. A few computational tools have begun to emerge (cBioPortal [11], COSMIC-3D [30], CRAVAT [31], Jalview [32], MuPIT [33], MutDB [34], STRUM [35], Cancer3D [36]) that enable users to take individual genetic variations, or a list of them, and visualize these in the context of protein structures. For example, CRAVAT [31] allows a user to upload a variant call format (VCF) file [37] (a file format used for representing DNA sequence variations) containing many genetic variants and assess which of those variants map to proteins, and then to explore individual variants in a 3D visualization of each protein when available. STRUM [35] allows users to visualize the structural model of a protein while, in addition, providing the profiles of the folding free-energy changes induced by the single-nucleotide polymorphisms (SNPs) or mutations. The starting point of STRUM is the wild-type sequence with SNPs or mutations, whereas I-TASSER is used to generate 3D protein models from which the impact of genetic mutations on protein stability can be more accurately calculated compared with the sequence-based approaches. Other tools, such as Jalview [32], provide a workbench for exploring variants in context with multiple sequence alignments, molecular structures, and annotations. COSMIC-3D and cBioPortal [11] map and visualize variants in their databases on 3D protein structures. The VIPUR pipeline [38] goes one step further and allows automatic interpretation of the effect of the mutation on the protein structure. The input to VIPUR is the wild-type sequence and the mutation of interest, and, based on the availability of a known structure or homology model, the tool maps the mutation onto the structure, and uses Rosetta [39] energy terms (Box 1) as indicators to report which features are most strongly affected by the mutation. Broad mining of data across thousands of proteins and millions of variants remains challenging due to the computational cost of structure modeling and the limited availability of experimental structures and high-fidelity models.

The confluence of genetic variation information and protein structure knowledge has broad applications across multiple fields of study, including precision medicine [40]. A future is conceivable in which an individual’s genetic variants are uploaded to an intelligent system that can flag variants for previously documented functional alterations, and then enable a clinician or genetic counselor to explore the potential implications for health and disease, based on the predicted effects of these variants on the functions of individual proteins. Similarly, decisions about which therapies are indicated may be influenced or directly based on the known function of a drug as it relates to potential variants on the drug’s target protein. Such a system remains distant, but the time is right for developing an infrastructure that would enable its development. There are a few ongoing efforts to curate functional data and disease associations for cancer variants [41–44]. Efforts to computationally model the association of various genomic mutations and human diseases are also underway [45–47].

Although the handful of tools listed above already perform an integration of genetic variation and protein structure data at some level, building infrastructure for both large-scale integration as well as broader usage of tools in the laboratory and in the clinic has yet to be achieved. Large-scale data integration for millions of variants, thousands of genomes, and tens of thousands of structures on platforms such as Apache Spark [48] and Google BigQuery [49, 50] will enable complex queries and machine-learning approaches to further learn how to predict functional implications of detected variants.

In order to accelerate progress in this field, we held a workshop on this topic at the Institute for Systems Biology in Seattle in February 2017. Here, we summarize the discussions and conclusions of this workshop, and present a comprehensive overview of the field. Finally, we conclude with a proposed architecture for a framework that could allow improved interoperability between the tools in this domain, making it easier for everyone to build on the accomplishments achieved so far.

## The gene variation to 3D workshop

On 9 and 10 February 2017, the Gene Variation to 3D (GVto3D) workshop was hosted at the Institute for Systems Biology in Seattle, Washington. The goal of the workshop was to explore the state of the field connecting genetic variation and 3D protein structure, and to bring together some of the key researchers working on interpreting genetic variation data. The workshop consisted of a mixture of talks, discussion sessions, and breakout groups. The program is available at the workshop website [51]. Twenty-five speakers provided short (15 minute) summaries of their research; highlights from the talks are available from the meeting website [51]. The oral presentations connected the workshop theme to diverse topics such as RNA sequencing (RNA-seq), big data technologies, how precision medicine can help with specific diseases, and cancer research.

After all the presentations and discussion sessions concluded, workshop participants separated into two breakout groups to brainstorm about how the research community as a whole could accelerate progress in the field in ways that individual laboratories could not.

Breakout group 1 discussed existing ontologies, tools, and datasets in the field and considered potential architectures for an integrative framework, focusing on how tools and resources could be made more interoperable to enable more widespread use of the tools and integration of inputs and outputs among the tools. Important aspects that emerged in the discussion include:

1. Adoption or development of standardized formats for the various major data types (such as variants, splice isoforms, post-translational modifications, structures, sequence annotations, and phenotypes).
2. Mechanisms to scale up the information exchange to large-scale queries using big data technologies such as DataFrames [52] and BigQuery [49].
3. Use of ontologies to standardize the terminology for the exchange of data and knowledge. These ontologies already mostly exist, and need only be specified as the standard, although some extension may be required.
4. Selection of initial tools that should be part of a pilot phase of the development and initial deployment of the interoperability framework.
5. Development of a tool registry and portal that would serve as a web-accessible resource for finding relevant tools, their inputs and outputs, and also reference data files that can be used to demonstrate and validate the tools and their interoperation.

Breakout group 2 discussed unmet needs, ranging from improvements in structural interpretation of splicing variants to more effective dissemination of knowledge to clinical geneticists, tumor panels, and the general public. Salient questions and points that were discussed include:

1. How to increase the actionability of variants observed in patients. Beyond facilitating access to knowledge on the structural impacts of variants, there is a need for a metric of confidence in the predicted impact. Gene-editing technologies are likely to enhance experimental studies of salient variants.
2. The need to recognize multi-variant interactions within single genes and proteins and mutation effects on protein–protein, protein–nucleic acid, or protein–ligand and drug interactions. Also, annotation of the context in which each variant could have an effect is important. For instance, information on cell types or cellular conditions in which specific interactions or protein complexes are formed, as well as annotation of epistatic relationships with mutations elsewhere in the genome, can help in interpreting a mutation’s influence on the cell.
3. How to improve the interpretation of variants affecting splicing. A proposal was made to create a mechanism for collecting donated RNA-seq data to derive a comprehensive set of splice variants and interpret them in the context of protein structure. It may also be useful to organize data on splice variants by type of alternative splicing (for example, exon swaps, intron retention, and coordinated inclusion of distant alternative exons [53], which are widespread in the human transcriptome and primarily affect protein coding exons [54]).
4. How to standardize annotation pipelines and data integration methods. It was recognized that this has already been partially solved independently by various teams, such as mapping genomic positions onto 3D structures (see “Current State of the Field”), so there would be a benefit from implementing an interoperation framework.
5. How to identify the target audiences. Scientists, tumor boards, clinical geneticists, developers of targeted drugs, patients, and lay people with an interest in genetic testing were all identified as possible audiences.
6. How to improve documentation and outreach. Suggestions included the development of documentation videos and tutorials, and contributing to Wikipedia sections describing the impact of variants on protein structure, building on current experience such as the Protein Standards Initiative [55] of the Human Proteome Organization.

The workshop has already begun to positively impact collaboration and interoperability in the wider research community. For example, an immediate outcome from discussions that occurred during the workshop was that links pointing researchers to the MuPIT resource [33] were added to the Kaviar database of human SNPs [13] and the PeptideAtlas database of proteins detected via mass spectrometry [56, 57], so that the variations in the latter resources can be depicted using the tools in MuPIT. Engaging members of the research community, as we have, will enable promising avenues for further work in this direction, including the design of a framework according to principles of user-centered design. Before laying out our vision for the framework, however, we first provide an overview of the field as it stands.

## The current state of the field

Here we review methods that use 3D structural information from the PDB to predict the effect of missense mutations; mapping other types of mutations (for example, insertions, deletions, splicing effects) remains an open challenge. In Table 1, we present an overview of six classes of prediction methods, summarizing the type of prediction and listing some of their limitations. We have then reviewed the literature and assigned methods to these classes. Additional file 1: Table S1 presents an extensive summary of over 30 such methods that have been published in the past decade, and have a current web presence as a web-based user interface, a web service, or a downloadable stand-alone application. In addition, we have captured tools that rely on sequence information only. Prediction tools are trained, tested, and validated on sets of reference proteins and their mutated forms (benchmark datasets). In Additional file 1: Table S1 we have included a list of benchmark datasets commonly used to train prediction tools.

**Table 1:** Classification of methods to predict the effect of missense mutations

Method type	Prediction	Limitations
Protein stability	Predicts the difference in unfolding free energy between wild-type and mutant protein	Considers only one possible mechanism that may affect the phenotype
Protein–protein/protein–nucleic acid affinity	Predicts the difference in the binding affinity between binding partners upon mutation	Small training datasets limit the scope of these methods
Protein–ligand affinity	Predicts the difference in ligand-binding affinity upon mutation	Small training datasets limit the scope of these methods
Phenotypic effect	Predicts the likelihood that a mutation is deleterious without considering a specific molecular mechanism	Except for Mendelian disease phenotypes, the phenotype may only be observed in a subset of the population (partial penetrance). Databases use different annotation practices and contain contradictory information for some mutations
Mapping and 3D visualization	Provides a 3D context of the site of mutation and may give atomic-level insight into mechanism of action	Visual approach is not suitable for automated whole-exome predictions
3D mutation hotspots	Clusters mutations by spatial proximity that are not necessarily close in protein sequence	Clustering may not explain the effect of specific mutations in a hotspot

*3D* three-dimensional

A first set of methods predicts thermodynamic properties related to mutations: (1) change in protein stability [35, 58–72]; and (2) change in binding affinity for protein–protein [66, 73–78], protein–nucleic acid [66], and protein–ligand complexes [79]. These methods have been trained on data from wild-type and mutant protein pairs, often using protein stability data from the ProTherm database [80], protein–protein binding affinities from SKEMPI [81], protein–nucleic acid binding affinities from ProNIT [80], and protein–ligand binding affinities from Platinum [82].

A second set of methods [38, 58, 76, 83–88] predicts the phenotypic effect (pathogenicity) of mutations, most often as a binary classification: deleterious or neutral effect. These methods have been trained on data resources that either contain mostly germline mutations, such as ClinVar [14], HGMD [15], and OMIM [89], or somatic mutations, such as the Cancer Genome Atlas (TCGA) [90] and COSMIC [10]. Carefully selected benchmark datasets to develop and test prediction methods have been collected: VariBench [91] and VariSNP [92].

Few prediction methods are purely based on 3D structural information, with the exception of FoldX [63], which uses an empirical scoring function to predict the change in protein stability or protein–protein binding. Most methods (Additional file 1: Table S1) use a combination of structural and sequence features and then formulate a regression problem to predict scalar values (for example, affinity changes), or a classification problem to predict a mutation as probably deleterious or neutral. Some methods use homology models to increase structural coverage, when experimentally determined structures are not available. The use of structural information varies from method to method. FoldX uses the 3D atomic coordinates of the protein, whereas most methods extract structural features that characterize changes in the local environment around a mutated residue [38].

Most tools to predict the effect of mutations are available online. However, there is a wide variety of input formats and scope of prediction (that is, predicting the effect of a single or multiple amino acid mutations). The majority of the 3D protein structure-based tools take PDB residue numbers of the mutated sites as input (Additional file 1: Table S1). There are also tools that exploit structural models predicted by advanced structure modeling algorithms and demonstrate the usefulness of structure predictions compared to those using only sequences, such as FoldX [63] or BindProfX [78]. A smaller number of tools use UniProt/Swiss-Prot residue positions. A minority of tools use chromosome position, dbSNP ID [8], or VCF files as input. A few tools need explicit PDB structures in the wild-type and mutated forms. User interfaces and presentation of results with the available web resources vary significantly; some resources require a user registration, and in some instances results are returned by email.

Several integrated tools have been developed that combine the prediction of the effects of mutations, annotation by functional information, and visual mapping of mutation sites onto 3D protein structures and multiple sequence alignments. Examples include 3DHotspots.org [93], cBioPortal [11], COSMIC-3D [10], CRAVAT [31], Jalview [32], LS-SNP/PDB [94], MOKCA [95], MuPIT [33], RCSB PDB [21], SNP2Structure [96], and Cancer3D [36]. These tools might help elucidate the effect of mutations in the context of both 3D structure and other available annotations. Ensembl’s Variant Effect Predictor (VEP) [97] combines several annotation and prediction services, including various considerations of effects on protein products.

A biologist who wants to assess the effect of mutations is confronted with a bewildering set of tools and options. The high variability in the user interfaces and in the representation and retrieval of results makes a systematic comparison of predictions by multiple tools cumbersome and requires manual input; hence, most tools are not applicable to anything but a small set of selected mutations. A systematic or automated comparison of a list of mutations (for example, at exome scale) using multiple tools is generally not possible. For instance, it would be useful to run tools that predict multiple effects of mutations simultaneously, such as protein stability and interruption of protein–protein and protein–nucleic acid binding. A further limitation is the input by PDB or UniProt residue position, since SNVs are annotated using genomic coordinates. Mapping between genomic and protein coordinate systems is error prone due to, for example, different genome assembly versions and alternative splicing. Where a mapping from genome to UniProt is possible, SIFTS [98] and CRAVAT [31] provide consistent residue-level mapping to and from PDB structures and other resources.

Current tools that predict the effect of missense mutations are based on either protein sequence information, 3D structural information, or both. Tools predict either biophysical changes or effect on phenotype. Those that use 3D structural information and visualization offer additional insights by providing locations of mutations in a 3D context, which is not possible using sequence-based prediction. For example, multiple mutations on a protein can be visualized and potential 3D hotspots can be identified. In the next section, we describe a framework to overcome the large heterogeneity of tools, which limits their usefulness, ease of use, and hinders comparative performance assessments.

## Proposed framework for making progress as a community

To facilitate innovation in this field, we recommend the development of a framework of common formats and application programming interfaces (APIs) that enable the many resources available to interoperate more effectively both at the individual variant level and at large scales. We further recommend the development of a portal that can be used to annotate the current state of tools in the field and guide users on how these tools can interoperate and be used to address different research questions. The outline of the recommended GVto3D framework takes its lead both from our wider review of the field as well as from the presentations and discussions that occurred among those members of the research community who attended the workshop; its design incorporates the needs and existing efforts of these researchers.

**Fig. 1:**
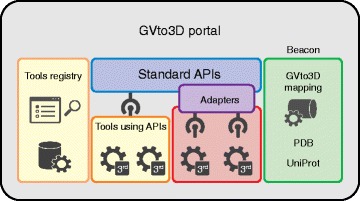
Components of the GVto3D portal. The Tools Registry contains a searchable description and metadata for tools, resources, and reference data sets for third-party variant effect prediction and annotation services. Standardized application programming interfaces (*APIs*) provide interoperability for data input and output of these third-party tools. Custom adapters can provide limited interoperability for tools that cannot adopt the API. A mapping service provides bidirectional mappings from reference genome coordinates to UniProt protein positions and to Protein Data Bank (*PDB*) residue positions. The tools can use the mapping service to accept variant positions in any of the three coordinate systems. A beacon system enables queries about variant positions where three-dimensional (*3D*) structural information and annotation are available

Figure 1 depicts the recommended components and design of the GVto3D framework. The Tools Registry will act as a central repository of data resources and software tools related to genetic variants, protein sequences, protein structures, variant effect prediction, and variant annotation. Metadata about each resource to enable findability of the different software tools will be stored and offered through an interactive web interface and also an API, which in turn enables the development of intelligent software that can automatically discover applicable resources and gather information about how to communicate with them to obtain the desired results. In addition to name, description, citations, contact information, and uniform resource locators (URLs), each entry will contain information important to the tool’s interoperation, such as the inputs and outputs, API support, and reference genome information.

A second component of the portal will be the definition of standard APIs so that information can be sent to and requested from different tools in the same way, thereby reducing software development overheads, which are typically encumbered with different tools using different APIs. It is envisaged that new third-party tools will use the API natively while API adapters will be developed in order to bridge with pre-existing third-party tools. The API enables seamless interoperability between different variant-related tools and also a standard access to multidirectional mapping among genomic, protein sequence, and protein structure coordinates. These mappings will be made available through APIs and as downloadable data files. Mappings will be kept up to date based on the update schedules of the underlying data sources (PDB, weekly; UniProt, monthly), freeing developers from maintaining and updating copies of these data. Once several similar resources support the standard APIs, the site can be further developed into an aggregation portal, where a query at the portal can be automatically farmed out to multiple resources, and the results collated and returned to the user in a single batch. This framework advances the FAIR principles of findability, accessibility, interoperability, and reusability [99] for all tools and resources that participate.

The use of standard file formats and standardized representations of data enable interoperability of prediction tools, for example, the output from one tool can be passed as input into a second tool, and can thereby simplify the comparison of different methods. The standardized formats are also essential components of a reusable set of integrated tools (software stack), including tools for reading and interpreting data files (file parsers), APIs, and visualization tools. Most of the current tools use a variety of inputs and outputs, placing a large burden on the user to transform data. Standard file formats and uniform APIs will be at the core of future services that will combine and compare different approaches. Various platforms and tools have different schedules and reliability of upgrades; keeping track of versions is important as changes to software may have large effects on the results.

The VCF file format [37], despite its complexity, is the de facto standard format for storing variant calls for a wide range of variants, from SNVs to long insertions and deletions. The Global Alliance for Genomics and Health’s Data Working Group File Formats Team defines the VCF specification and its evolution [100]. Variant annotations—for example, the results of prediction tools—can be captured in the INFO records, which are a set of structured records used to add annotation to VCF files. VCF versions 4.x, including the current version 4.3 [101], define meta-information lines that describe the INFO record data types and enforce standardization [102]. In addition to VCF, a few other formats have been described, such as ANN, which defines a different standard for representing variant information in INFO fields; VEP [97] supports a simple tab-delimited, as well as JavaScript Object Notation (JSON) output format.

Regarding genome nomeclature, the Human Genome Variation Society, which aims to foster the discovery and characterization of genomic variations, including population distribution and phenotypic associations, has established guidelines and recommendations for the nomenclature of gene variations, and serves as an international standard [103].

Progress in this field depends on global collaboration and the sharing and reuse of tools. APIs provide protocols to enable this collaboration. Tools wrapped in standard APIs present a consistent interface to heterogeneous tools, enhancing interoperability, and shielding the user from changes to the underlying software. As an example, many prediction tools that use 3D protein structural information define the location of mutations at the protein level using either UniProt or PDB coordinates. Mapping genomic coordinates to 3D protein structure is non-trivial and error prone. Robust APIs that can perform this mapping with up-to-date 3D information using both types of protein coordinates can augment existing tools that are based on just linear protein sequence coordinates.

Moreover, progress in the prediction of the effect of mutations and use of 3D structural information depend on the availability of well-designed training, test, and validation sets. The tool repository will be a place to share datasets, as well as protocols and references (metadata) for how these datasets were generated. Validation sets, accompanied by well-documented tutorials or vignettes, will include a subset of variants with clearly understood effects that can be used to test the output of available resources. Eventually these can serve as a set of unit tests for the framework itself.

## Conclusions and future perspectives

The GVto3D workshop held in Seattle in February 2017 represents an important step towards spurring collaboration and advancing progress in proteogenomics research. The disparate nature of current tools and resources and lack of interoperability contribute to slower progress in the field than might otherwise be possible. Development of a community-driven interoperability framework for integrating genetic variation resources and protein structure resources promises further expansion of our understanding of the functional implications of genetic variation. While the use of 3D structural features has enabled the atomic-level exploration of the effects of mutations (for example, the identification of 3D mutation hotspots), the accuracy, scope, and scale of predictions are still limited. The proposed framework will enable pooling of data sources and tools, and collaborative development.

However, there will be substantial challenges as we move forward with design of the framework. The first challenge is establishing a durable user base for the framework. One possible approach is to engage a few key laboratories to take the lead as early adopters, and assume that the framework will gain wider community acceptance through their example. We propose a more user-centered design approach, however, which emphasizes an iterative process of engaging multiple communities of practice in conceptualizing, developing, and rolling out the framework; the GVto3D workshop was a first step in this direction. Second, questions of sustainability are also pertinent here, insofar as how such a system will be maintained, and who will be responsible for its ongoing maintenance. Here, we propose an approach that we envision will become self-sustaining through the deployment of open-source technologies in an engaged community. Third, standardization is a key component of any interoperability project, which in this case depends upon work to enhance usage of certain de facto standards, and to establish other standards, including the creation of standard APIs. Working closely with the community of potential framework users, as well as with standard-setting bodies, such as the Global Alliance for Genetics and Health and the Proteomics Standards Initiative of the Human Proteome Organization, will be important for helping these standards gain further traction.

Taken together, the user-centered framework we have outlined above—a Tool Registry and a set of standardized formats and common APIs based on deployment of open-source materials—aims to bring the FAIR principles to bear on current and emerging tools while enabling their broader usage across multiple communities of practice. The result promises to be more rapid progress in research that can make use of GVto3D resources and eventual applications to precision medicine, while ensuring that methods and outcomes are findable, accessible, interoperable, and reusable.

## Box 1. Glossary

Benchmark dataset: A curated and well-studied dataset that can be used to evaluate the relative performance of analysis methods and algorithms.

File parser: A computer program module that interprets the structure of input data and breaks the input into well-defined parts that can then be used by other parts of the computer program.

Rosetta energy terms: Rosetta [39] estimates the energetic stability of protein structures as a sum of energy terms, including hydrogen bonding, electrostatic interaction, attractive and repulsive interaction, and solvation terms.

Software stack: A set of software subsystems or components designed to work together as a platform.

Variant Call Format: A standard format of a text file used for storing genome sequence variations relative to a reference genome.

## Additional files

Additional file 1: Table S1. shows tools that use three-dimensional structural information from the Protein Data Bank to predict the effect of missense mutations. (XLSX 43 kb)

## Abbreviations

3D: Three-dimensional
API: Application programming interface
FAIR: Findable, accessible, interoperable, reusable
GVto3D: Gene Variation to 3D
JSON: JavaScript Object Notation
PDB: Protein Data Bank
RNA-seq: RNA sequencing
SNP: Single-nucleotide polymorphism
SNV: Single-nucleotide variant
URL: Uniform resource locator
VCF: Variant call format
VEP: Variant Effect Predictor
VUS: Variant of uncertain significance
